# Reflection of treatment proficiency of hydroxyurea treated β-thalassemia serum samples through nuclear magnetic resonance based metabonomics

**DOI:** 10.1038/s41598-019-38823-0

**Published:** 2019-02-14

**Authors:** Ayesha Khalid, Amna Jabbar Siddiqui, Saqib Hussain Ansari, Syed Ghulam Musharraf

**Affiliations:** 1 H.E.J. Research Institute of Chemistry, International Center for Chemical and Biological Sciences, University of Karachi, Karachi, 75270 Pakistan; 2 Dr. Panjwani Center for Molecular Medicine and Drug Research, International Center for Chemical and Biological Sciences, University of Karachi, Karachi, 75270 Pakistan; 3 Department of Pediatric Hematology & Molecular Medicine, National Institute of Blood Diseases and Bone Marrow Transplantation, Karachi, 75300 Pakistan

## Abstract

β-Thalassemia is a widespread autosomal recessive blood disorder found in most parts of the world. Fetal hemoglobin (HbF), a form of hemoglobin is found in infants, replaced by adult hemoglobin (HbA) after birth. Hydroxyurea (HU) is one of the most effective HbF inducer used for the treatment of anemic diseases. We aimed to improve the understanding of HU therapy in β-thalassemia by metabonomics approach using ^1^H NMR spectroscopy. This study includes 40 cases of β-thalassemia before and after HU therapy along with 40 healthy as controls. Carr-Purcell-Meiboom-Gill (CPMG) sequence was used to identify forty-one putative metabolites. Generation of models like partial least square discriminant analysis (PLS-DA) and orthogonal projections to latent structures discriminant analysis (OPLS-DA) based on different metabolites including lipids, amino acids, glucose, fucose, isobutyrate, and glycerol revealed satisfactory outcomes with 85.2% and 91.1% classification rates, respectively. The concentration of these metabolites was altered in β-thalassemia samples. However, after HU treatment metabolic profile of same patients showed closeness towards healthy. Deviant metabolic pathways counting lipoprotein changes, glycolysis, TCA cycle, fatty acid and choline metabolisms were identified as having significant differences among study groups. Findings of this study may open a better way to monitor HU treatment effectiveness in β-thalassemia patients, as the results suggested that metabolic profile of β-thalassemia patients shows similarity towards normal profile after this therapy.

## Introduction

Each year approximately 300,000 children are born with hemoglobinopathies, like thalassemia and sickle cell anemia^1^. Thalassaemias are autosomal recessive blood disorders that can be divided into alpha (α) and beta (β) thalassemia. The prior results from abnormality of α gene positioned on chromosome 16 while mutation on the chromosome 11 cause β- thalassemia with either absent or decreased synthesis of the β-globin chain, a constituent of adult hemoglobin (HbA)^2^. Imbalance in α/β-globin ratio causes oxidative damage to membrane lipids and proteins of red cell triggering their destruction and eventually anemia and its related complications^3,4^. A number of strategies are in practice for the treatment of β-thalassemia which includes transfusion therapy, iron chelation therapy, fetal hemoglobin (HbF) induction, gene therapy and hematopoietic stem cell transplantation (HSCT). HbF, a form of Hb tetramer found in infants, contains two α and two γ (gamma) chains^5^. Its production is replaced by HbA around six months postnatally^6^. Induction of HbF as a substitute of HbA in order to treat anemic diseases is an exciting treatment options^7^. Among various identified therapeutic agents for γ-globin gene induction^8–12^, hydroxyurea (HU) has earned extensive approval by FDA to be used as HbF inducing agent^13^. Hydroxycarbamide or HU is an antimetabolic, cytotoxic and antineoplastic agent used for myeloproliferation disorders. However, it also causes HbF induction and the mechanisms by which HU induce HbF are unclear^14^. At this time, more than 200 mutations have been found in the β-globin gene and linkage of few of them with response to HU has also been reported^15–17^.

By examining bio fluids particularly, changes in metabolites involved in different biochemical processes provide a more functional manifestation of a biological system. Metabonomic profiling techniques like NMR in combination with chemometric method render a gateway to examine these changes in small molecules that are associated with disease prognosis and recovery after treatment. NMR based metabonomic studies for evaluation of drug efficacy on different diseases by models have already been done^18–20^. It can be postulated that in thalassemic patients after treatment with HU may result in changes in their gene expression that ultimately induce an alteration in the metabolome of patients^21,22^. Also, an attempt was made in past to relate NMR based findings with hematological parameters like HGB (hemoglobin), MCH (mean corpuscular hemoglobin), CT (hematocrit) and some others of healthy and thalassemic patients^23^. But so far, no such report by the scientific community has focused on an association of β-thalassemia patient’s metabonomics to HU treatment in order to reveal recovery status of patients from disease condition.

This study, was designed to fill this space by focusing on the untargeted metabonomic investigation of β-thalassemia samples treated with HU for HbF induction by means of NMR and present an unbiased information about metabolite profiles of HU- treated and untreated groups with healthy. That will ultimately assist to identify the differential metabolites that were responsible for discrimination of samples of three groups as well as to identify the effect of HU therapy on metabonomics of these patients. Several influential data mining and statistical bioinformatics methods were used for the identification, prioritization, and classification of robust and generalizable metabolites with high discriminatory ability. Furthermore, it would be expected that findings of this report play a pivotal role in strengthening the knowledge of metabonomics area of β-thalassemia. The information obtained in this study may help in future in investigating HU treatment effects in patients. Moreover, by in sighting into the metabolic patterns of untreated, treated and healthy one’s information about treatment efficacy and patient’s recovery position could also be accomplished by following this approach.

## Material and Method

### Patient’s Recruitment

The identification of patients for current research was executed in National Institute of Blood Disease and Bone Marrow Transplantation (NIBD), Karachi, Pakistan after the proper settlement of the Institutional Review Board (IRB)/Ethic Committee of the hospital and Independent Ethic Committee (IEC) of the primary investigating institute. All methods were performed in accordance with the relevant guidelines and regulations approved by the committee. This examination encompassed 40 follow up subjects of β-thalassemia and the blood samples of these subjects were collected before beginning of HU treatment and after HU treatment with following consideration criteria: Patients have been registered at NIBD, identified as β-thalassemia major case (characterized as Hb <7 g/dL, high HbF, a very low or absent HbA. These patients also require more than 8 transfusions per year. The diagnosis of β-thalassemia major will also be affirmed by the analysis of DNA where required). Sampling of these patients was carried out after 4–8 hours fasting state, before blood transfusion if patient requires it. Rejection criteria: Those patients were excluded from the study who had indicated any chronic diseases which are not related to β-thalassemia. Besides this, patients with hypersensitivity to HU were also excluded from the study. The HU treatment was given for at least 6–12 months, at a mean dosage of 16–20 mg/kg/d. This treatment was continued or discontinued after 6–12 months, after complete examination of the patients’ response to the drug. Blood from healthy subjects were collected as control for this study at ICCBS (International Center of Chemical and Biological Sciences). Detailed information of healthy subjects and patients are compiled in the Supplementary Information (Table S1). A comprehensive questionnaire related to study was also filled from each blood donor (healthy and patient) after obtaining written informed consent from each donor.

### Sample Collection

Collection of blood sample from each subject of healthy was done, after 8 hours of fasting state, however collection of the sample from newborn and toddlers was done when they were in a need of meal. A gel-based BD^®^ vacutainer tubes (BD Franklin Lakes NJ, USA, REF: 367381), whose inner walls are coated with silicone for clot activation was used to collect around 5 mL of blood that was drawn from each healthy subject. For partition of serum, centrifugation at 4 °C for 10 min at 2000 rpm was performed. After this aliquots of serum were drawn and kept instantly at −80 °C freezer until further treatment of the sample.

### Preparation and Analysis of Samples

Before analysis, the frozen serum samples were thawed on ice and after that vortexed. 100 μL (0.5 mM) of DSS [(trimethylsilyl)-1-propane sulfonic acid sodium salt] (IS) and 300 μL of D_2_O was mixed with 200 μL of the homogenized serum. Afterward, the mixture was vortexed for 60 secs, then centrifuged at 11,000 rpm for 5 min. 5 mm NMR tube was used for shifting of 550 μL of this mixture. Bruker Avance 500-MHz spectrometer (Bruker Biospin, Rheinstetten, Germany), outfitted with a z-gradient probe at 310.0 K was used to obtain ^1^H NMR spectra. 1D experiment was obtained for each sample. T_2_ edited ^1^H NMR spectrum (CPMG) experiment with water suppression was performed to enhance visualization of compounds having low molar mass. A 4 second recycle delay, 32,768 data points, 10-kHz spectral width, a total 30 ms of spin-echo time, 362 receiver gains, 16 dummy scans and a total 120 scans were used to attain spectrum. TopSpin 2.1 (Bruker Biospin, Germany) was used for manual correction of phase and baseline of acquired NMR spectra. DSS signal at (δ 0.00) ppm was adjusted as a reference for all chemical shifts.

Notwithstanding to this experiment, a few 2D experiments were additionally performed, that encompasses ^1^H-^13^C hetero nuclear single-quantum correlation (HSQC) spectroscopy, ^1^H-^1^H correlation spectroscopy (COSY), and ^1^H-^1^H *J*-resolved to confirm peak assignments to molecules in particular samples by means of previously reported literature^24,25^ and existing databases^26^.

### Chemometric Analysis

Reduction of spectral region of *δ* 13 to −2 of each spectrum (over-all 120 spectra) into rectangular buckets having a width of 0.04 ppm was attained by AMIX version 3.9.14 (Bruker Biospin, Rheinstetten, Germany) after excluding the region having resonance of residual water (*δ* 4.66–4.90). Moreover, the exportation of above mention bucket tables on SIMCA-P package (version 14, Umetrics, Umeå, Sweden) was done. Normalization of all of the data to the unit area was performed (to expel alterations in concentration among metabolites), providing equal weight in face of absolute value to all variables. The normalized data, Gaussian curve shown in Fig. S1, was log transformed and Pareto scaling algorithm was employed and then auto fit for multivariate analysis.

Initially, an overview was generated by performing principal component analysis (PCA) to get clustering behavior among groups and to exclude outliers. Execution of partial least squares discriminant analysis (PLS-DA) and orthogonal projections to latent structures discriminant analysis (OPLS-DA) assist to drop the inconsistency in intergroup, magnification of variances among the samples and to calculate variables that are responsible for classification. The variable loading plot from a validated OPLS-DA model was ranked with regard to their performance of discrimination among groups. This was achieved through the variable importance in projection (VIP) values. The 7-fold internal cross-validation as a default method of the software was applied. For added validities of models, permutation methods and CV-ANOVA were also performed. To recognize the differences among groups of samples, the VIP list and the analogous loading plots of every model were prudently scrutinized and lastly used to ascertain which variables were imperative for refinement among the groups.

## Results

**Figure 1:**
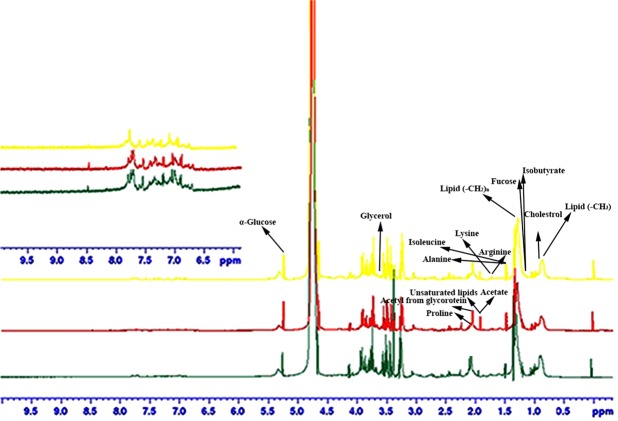
Stacked view of T_2_- edited (CPMG) ^1^H NMR spectra of blood serum from healthy (green), untreated (red), treated (yellow). The low field region (δ 6–9) is vertically projected 10 times relative to the rest of the spectrum.

**Figure 2:**
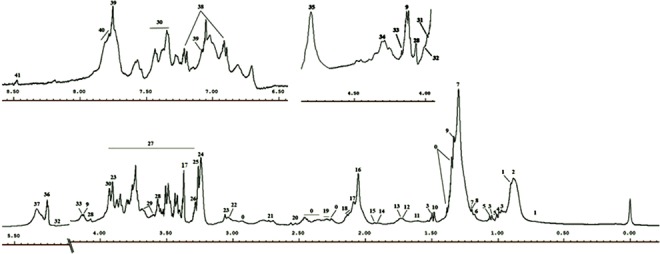
Assignments of the ^1^H NMR signals on the basis of 2D NMR experiments of a representative 500 MHz 1D-CPMG ^1^H-NMR average spectrum of a healthy sample measured at 310 K. A, full spectrum (δ 5.50–0.5 ppm) and magnification of aromatic region (δ 9.00–6.50 ppm) and (4.00–4.50) region. Peak assignments: 0, unidentified; 1, cholesterol; 2, lipids (–CH_3_) (mainly LDL/VLDL); 3, isoleucine; 4, leucine; 5, valine; 6, isobutyrate; 7, lipids (CH_2_)n (mainly LDL/VLDL); 8, fucose; 9, lactate; 10, alanine; 11, adipicacid; 12, arginine; 13, lysine; 14, acetate; 15, lipids (CH_2_–C=C); 16, acetyl signals from glycoproteins; 17, proline; 18, glutamine; 19, lipids (CH_2_–CO); 20, citrate; 21, lipids (CH=CH–CH_2_–CH=CH–); 22, Albumin lysyl; 23, creatine; 24, choline; 25, taurine; 26,trimethyl N-oxide; 27, glucose and α-protons of amino acids; 28, Myo-inositol; 29, glycerol; 30, phenylalanine; 31, creatinine; 32, glycerol of lipids; 33, 3-hydroxybutyrate; 34, threonine; 35, β-glucose; 36, α-glucose; 37, lipids (–CH=CH–); 38, tyrosine; 39, histidine; 40, 1-methylhistidine; 41, formate.

120 serum samples from the all groups (40 for each group healthy, treated and untreated) were involved in this study. Figure 1 spectacles piled vision of average spectra of each group by means of CPMG experiment sequence; the signals in lower and upper field region comprising low molar mass metabolites differed in their intensity in respective groups. The consigned signals were mainly build on an average spectrum by the usage of CMPG sequence as stated in the literature^24,25^ and online databases (Fig. 2). ^1^H and ^13^C chemical shifts of identified metabolites were mentioned in Table S2. Amino acids, glucose, organic acid, sugars etc are few foremost, representative assigned metabolites that exist in serum. Fatty acyl chain of glycoproteins and lipoproteins produce many resonances that visualized from the CPMG spectrum. Visual valuation of all these spectra bared a vibrant increase/decrease within the concentration of all metabolites in untreated and HU treated in contrast to healthy controls.

### Statistical Analysis

**Figure 3:**
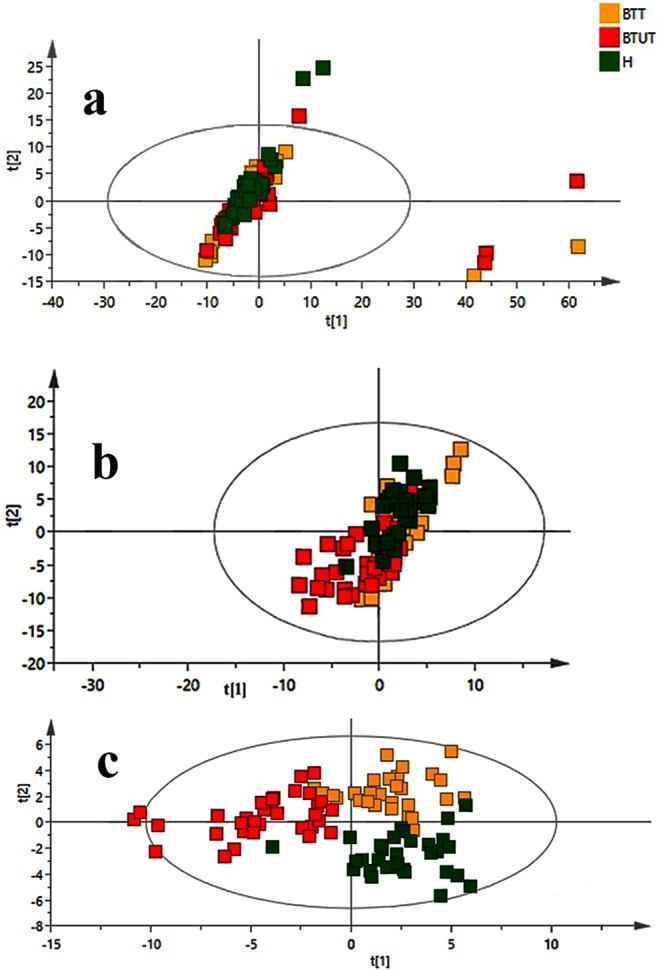
Scores scatter plots (**A**) PCA, (**B**) PLS-DA and (**C**) OPLS-DA of ^1^H CPMG NMR spectra of serum from Healthy (green), Treated (yellow) and Untreated (red) β-thalassemia samples.

Multivariate analysis was executed on the data to find out the metabolomic pattern among healthy, treated and untreated samples. Initially, PCA analysis was implemented in command to achieve a tendency of the parting of samples according to groups (Fig. 3a). Another PCA study was also conducted to see dependency of metabolic profile on age of subjects (Fig. S2). On no account variances associated with gender and age were originate in PCA analysis, though eight samples exhibited sturdy outlier behavior and lay outside the Hotelling’s 99% confidence limit (Fig. S3). A comprehensive examination of the mentioned samples grounded at the clinical analysis of these patients exposed the fact that either they had extremely disruptive glucose and blood profile. Hereafter these outliers were then omitted from further analysis and PCA was regenerated to confirm the absence of outliers (Fig. S4), which showed only 2 samples outside the Hotelling’s 99% confidence limit. For further separation and to attain more pronounced discrimination among groups, PLS-DA and OPLS-DA models were also performed, for further separation of groups. Figure 3b, score plot presented slight separation of groups, though Fig. 3c revealed intelligible parting among the groups. The summarized results of these models are presented in Table 1. A two group OPLS-DA comparison (Fig. S5) was also performed to insight into clustering of group and to discover latent metabolites that are responsible for group separation. An S-plot corresponding to every OPLS-DA was also presented in the figure, which showed that variables related to the ppm values of β-glucose are the most reliable variables for discrimination of two groups. Using the same two groups’ combination, volcano plots with p-value generated by Wilcoxon Rank test were made (Fig. S6). The significant variables obtained from volcano plots are the same from the S-plot that are mainly sugar, more specifically β-glucose. However comparing treated and treated cases ends up with unidentified variables.

**Table 1:** Average prediction results obtained by a default method of 7-fold internal cross validation of the software for PLS-DA and OPLS-DA models based on CPMG spectra of serum from Healthy, Treated and Untreated β-thalassemia samples.

Model	R2	Q2	Sensitivity	Specificity	Classification rate
PLS-DA	0.525	0.293	80.17%	82.99%	85.19%
OPLS-DA	0.81	0.513	90.78%	91.66%	91.07%

### Validation of Supervised Models

**Figure 4:**
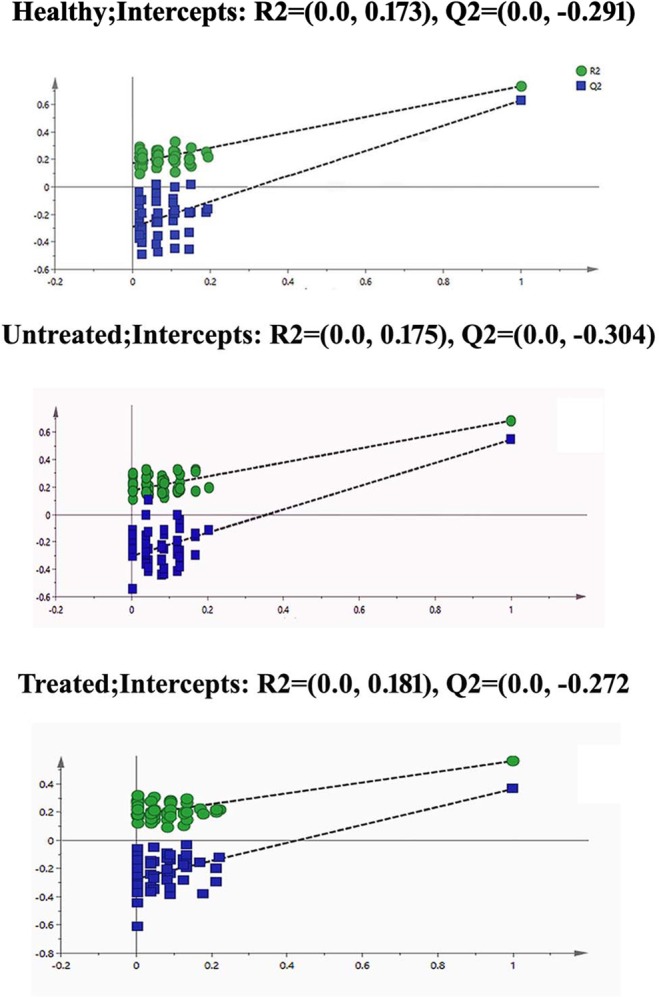
Permutation plots for the OPLS-DA model showing R2 (green) and Q2 (blue) values.

Two validation methods permutation tests (Fig. 4) and CV-ANOVA (Table S3) were performed on OPLS-DA. Permutation tests are performed to check the predictive ability of the model and tell how much risk to have a false model. A false model means that it can only fit to the training set accurately. For generating permutation plots, X-observations are remain the same while the Y-observations are randomly permuted to several new models. The goodness of fit parameters, that are R2 and Q2, from original and permuted models are then correlated to have a permutation plot. For a strongly validated model, the model points at right should be at higher level than Q2 values at left. Another interpretation of good permutation plot is that the regression line of the Q2-points should intersect to the vertical axis at or below the zero. Both of these interpretations were best fitted on the permutation plots of the OPLS-DA model of our study.

CV-ANOVA estimates independent predictors or OPLS-scores using the cross-validation of predictive residuals. It gives more reliable results as compared to simple ANOVA. The p-value suggests the model validity; the lower the p-value, the higher the separation between classes. Another validation tool of model is receiver operating characteristics (ROC) curve and area under the curve (AUC) values. This curve is plotted as 1-specificity vs sensitivity. Specificity is the ability of a model to predict samples from the class of controls, while sensitivity is to correctly classify samples of the class of cases. The AUROC values range from 0 (0.5 and lower usually means no discrimination at all) and 1 (perfect discrimination between classes).

In this study, the sensitivity and selectivity values of the model for healthy control group is 99.4% although the values for AUC for other groups were found to be 0.95, 0.96 for treated and untreated, respectively (Fig. S7).

### Distinguishing Metabolites for Treating β-thalassemia

**Figure 5:**
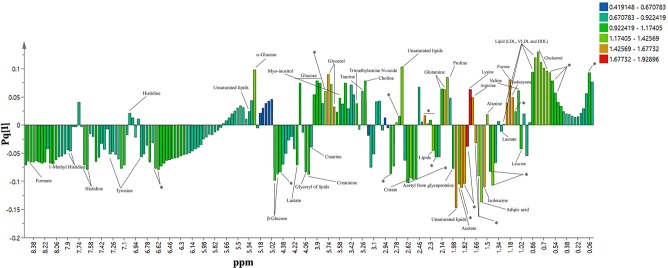
OPLS-DA loadings plot colored as a function of VIP. Assignment of main signals having values (1–2) is indicated (unassigned signals with high VIP are marked with an asterisk).

**Figure 6:**
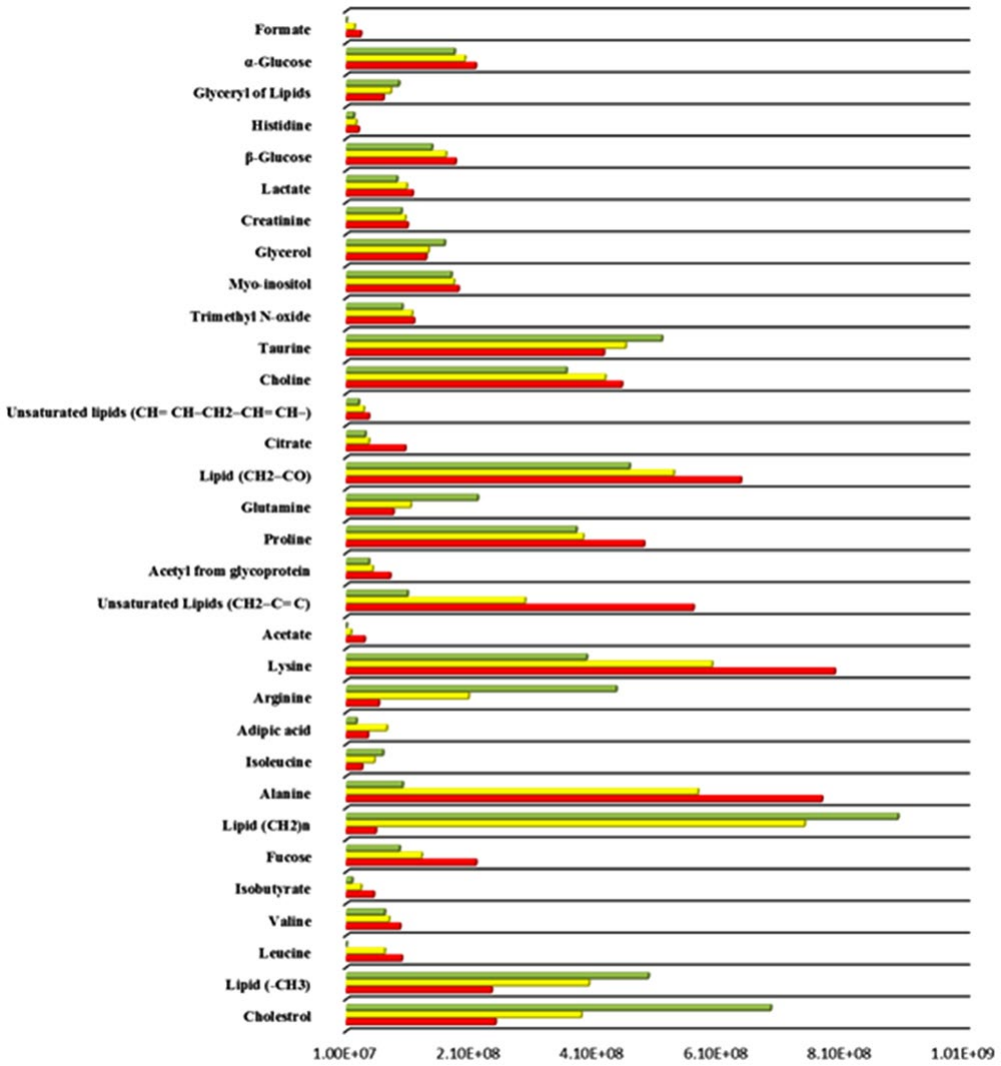
Average changes of main metabolites contributing to the discrimination between serum of thalassemic patients and of healthy subjects [Healthy (green), Treated (yellow), Untreated (red)].

For highlighting metabolites that are responsible for class separation in OPLS-DA model, combine loading plot and bi loading plots were colored as a function of VIP values > 1 (Fig. 5 and Fig. S8). After color coding using VIP values, high VIP points are also labeled to metabolites name. Considering combine plot mainly lipids appeared at different regions of spectra. The positive and negative signals of lipids are probably due to the splitting of fatty-acyl chain resonances, and HDL and LDL + VLDL lipoprotein are the lipids in this splitting pattern. This hypothesis was confirmed by presence of choline at 3.22. With regard to low molar mass metabolites, the OPLS-DA loadings with high VIP values (>1.0) were because of glycerol, few amino acids like (histidine, alanine, valine, lysine, proline, glutamine, and arginine), formate, acetate, citrate, adipic acid, fucose and glucose, signifying their state of prominence between healthy control and disease, when weighing up their concentrations. Moreover, few additional signals designated high VIP values, can be acclimated for sample discrimination in both (low and high) frequency regions (indicated in Fig. 5 with asterisks). In view of bi-loadings we get different set of metabolites that are responsible for discrimination of respective groups. Like VIP plot between BTUT (β-thalassemia untreated) and healthy comprises different classes of lipids and amino acids, glucose, glycerol and acid, a similar trend of metabolites is followed in discrimination of BTUT and BTT (β-thalassemia treated) groups. While plot between healthy and BTT showed different set of metabolites that include amino acid, glucose and glycerol only. A few other signals were also observed that marked with asterisks in all three plots. Though, identification of these asterisks signals at this stage was not performed. To confirm the alterations accentuated by loadings examination, CPMG spectra have been integrated, and the mean areas of particular signals among control and treated and untreated groups have been compared. Figure 6 gives a sound reflection of variation among certain metabolites that discriminate treated, untreated, and healthy control serum samples. The signals that used to generate a comparative profile of different identified metabolites are represented in bold form in Table S2. It was revealed that apart from few metabolites, the concentrations of rest of the metabolites were increased in the other disease groups. ROC plot was also produced for few selected discriminating metabolites (Fig. S9). In comparison between treated and untreated cases, isoleucine and unsaturated lipids gave the highest AUC values while lysine and arginine gave high AUC when diseased samples were compared with healthy one.

## Discussion

β- Thalassemia is an extremely pervasive (spread widely throughout an area or a group of people) genetic hematological disorder. Different challenges are faced by therapeutic centers in the whole world to deal these patients, because they not only demand a regular blood transfusion but also an iron chelation therapy to avert iron overload. Surrounded by many treatment strategies HbF induction is one of the finest choice in this regard. And among all HbF induction agents, HU is one of the most promising option in this esteem, because in one hand it reduces blood transfusion, on the other hand, it reduces other related complications too. The effectiveness of HU is still uncertain and need further research in this realm to relate its role in the management of the disease. Examination of genetic variation due to HU treatment has been done^27^, but metabonomics response to HU treatment remains an open area for investigation. For pathological diagnosis; prognosis and recovery high-resolution ^1^H NMR spectrometry for biofluids metabolomics is a less invasive high potential technique. Therefore, we targeted metabolome of β-thalassemia patients to study disease prognosis, recovery from disease state after medication with HU to induce HbF for 6–12 months.

Noticeable variations of serum metabolites was observed after the evaluation of metabolic profile of HU-treated, untreated and normal subjects groups. On the whole, HU-treated group’s metabolic profile as compare to their untreated samples has a shift towards healthy controls. The zoomed image of CPMG spectra revealed forty-one (41) metabolites wherein the metabolites that are identified are specified by their name while unidentified with zero. The OPLS-DA loading plot examines significant metabolites among these identified metabolites. The average bar graph is actually a reasonably clarifying evidence of difference in intensities of metabolic profile of untreated group from HU-treated and the control group, and an increase in concentration of some metabolites was also observed in the samples even though of few is decreased. It was proclaimed in literature that there is disturbance in metabolism of β-thalassemia patients^28^, that is also confirmed by the shift in metabolic profile of untreated patient from normal sera profile. On the other hand, HU treated sera profile of patients reflects the improvement in metabolic profile. Therefore, knowledge associated with these altered metabolites plays an imperative part in understanding of disease progression, recovery and efficacy of medication.

β-thalassemic patients have impairment in glucose homeostasis^22^. The occurrence of such endocrine disorders in β-thalassemia patients relates to a number of factors which may include an impaired β-cells function that in turn reflect a reduction in insulin secretion index, age, the severity of genotype, annual red blood consumption, uncontrolled iron overload, number of blood transfusions and few others^22^. HU treatment would probably resolve these complications and bring glycemic profile back to normal, as indicated in the result. Appropriate insight into the glycemic abnormalities in patients direct in early diagnosis and management of the disease. One of the principal element of triglycerides and phospholipids is glycerol that can be utilize as a source of energy after conversion in to glucose by liver. It also involves in metabolism of glycerophospholipid and glycerolipid. Identification and quantification of abnormal level of glycerol in many disorders have been done^29,30^. So, it is certain that during β-thalassemia because of metabolic stress glycerol is more consumed for energy production. Serum glycerol levels were going back to the normal range after HU medication because of reduction of metabolic stress in β-thalassemia patients as less glycerol is consumed for energy production. Fucose is a hexose deoxy terminal sugar in glycoconjugates in mammals, insects and plants, fundamental sub-unit of fucoidan (sulfated) polysaccharide. Critically regulates various physiological and pathological phenomena, including cancer and inflammation. Currently, fucose gaining considerable importance in the post-genomics and post-proteomic period of diagnosis and prognosis of cancer^31^. Fucose containing sulfated polysaccharides (FCSPs) offers several potentially beneficial bioactive functions for the human. Former study reveals that high levels of it induce aggravate apoptosis may be accountable for early degradation of RBCs in β-thalassemia^32^. HU treatment decrease RBCs degradation resulting in the improvement in membrane integrity. Therefore, the level of fucose returned to normal post-treatment.

Alteration in lipid metabolism causes disturbances in different processes occur in the body that in turn cause development and progression of the disease. In β-thalassemia, liver damage accounts for the changes in lipid profile including cholesterol, HDL, LDL, and triglyceride. Lipid profile in patients with β-thalassemia revealed different level in different studies, suggesting variation in the factor influencing their levels in each patient^33,34^. It can be concluded from previous studies that an increased level of hemolysis in β-thalassemia may be a cause of disruption in phospholipids particularly phosphatidyl choline, phosphatidylethanolamine and phosphatidylserine in patients^35^. HU-treated patient’s metabolome profile of these showed closeness to the healthy controls that give a clear reflection of improvement in stated complications after treatment. Amino acids are nutrients and substrates for macro molecular synthesis. Amino acid metabolism may possibly be compromised in β-thalassemia and result in aberrant plasma amino acid concentrations, as more are redirected to the production of erythrocytes and increased secretion of them in urine^36^. Different amino acids show a different trend in their concentration abruption. Our observations as well consistent with previous observations^36^, few amino acids express amend behavior in our study. For example, in our case arginine concentration decrease in untreated as compare to other ones. This may be because arginine metabolism is associated with several pain mechanisms, as pain is the hallmark of the β-thalassemic condition and patients experience pain virtually all the time. Arginine metabolism affect other related amino acids as well, as it is a precursor of many other amino acids like proline, creatine etc.^37^. In general, we are able to affirm that after HU therapy metabolic stress decreases in β-thalassemia patients that results in the normal metabolism of several altered pathways.

## Conclusion

This study monitor alteration in metabolism due to genetic abnormalities, by examining disruption in serum metabolic profile of β-thalassemic patients (untreated) in contrast to HU (treated) and healthy group. Based on observations, it can be foreseen that High- resolution ^1^H NMR spectroscopy of bio fluids is an upright depiction of metabolic patterns and deliver information regarding the structure and composition of low molecular mass metabolites. It is likewise an impressive, powerful, and less invasive technique for inspecting disease states. Moreover, it has been found that HU treatment help in regressing the metabolic profile towards healthy. The metabolic pathways that involved in the segregation of these samples are glycolysis, lipoprotein changes, choline fatty acid metabolism, and protein biosynthesis. It will be thought-provoking to explore NMR based metabonomics in diagnosing phenotype of patients and improving and studying treatment options.

## Supplementary information

supplementary material
