# Supplementary material for: Reflection of treatment proficiency of hydroxyurea treated β-thalassemia serum samples through nuclear magnetic resonance based metabonomics
Source: Sci Rep. 2019 Feb 14;9:2041. doi: 10.1038/s41598-019-38823-0 (PMC6376050; doi:10.1038/s41598-019-38823-0)
Supplement: Supplementary file 1 — supplementary material [file 41598_2019_38823_MOESM1_ESM.doc]

**Reflection of treatment proficiency of hydroxyurea treated β-thalassemia serum samples through nuclear magnetic resonance based metabonomics**

Ayesha Khalid^a^, Amna Jabbar Siddiqui^b^, Saqib Hussain Ansari^c^, Syed Ghulam Musharraf^ab*^

*^a^H.E.J. Research Institute of Chemistry, International Center for Chemical and Biological Sciences, University of Karachi, Karachi-75270, Pakistan*

*^b^Dr. Panjwani Center for Molecular Medicine and Drug Research, International Center for Chemical and Biological Sciences, University of Karachi, Karachi-75270, Pakistan*

*^c^Department of Pediatric Hematology & Molecular Medicine, National Institute of Blood Diseases and Bone Marrow Transplantation, Karachi-75300, Pakistan*

*Corresponding author. Tel.: +92 213 4824924-5; 4819010; fax: + 92 213 4819018-9.

*E-mail address:* [musharraf1977@yahoo.com](mailto:musharraf1977@yahoo.com)

**Supplementary Table S1:** Age, gender and weight of study subjects

| **S.No.** | **Parameter** | **Follow up β -Thalassemia Patients on HU (n=40)** | **Healthy**  **(n=40)** |
| --- | --- | --- | --- |
| 1. | Gender(Male/Female) | 23/17 | 25/15 |
| 2. | Age(Years) | 9.93±6.68 | 18.73±7.93 |
| 3. | Weights(Kgs) | 23.53±12.78 | 47.375±16.34 |

**Supplementary Table S2:** Chemical shifts of Metabolites identified in 500 MHz 1D-CPMG ^1^H and ^13^C NMR spectra of human serum

| **Molecule** | **^1^H shift (δ)** | **^13^C shift (δ)** |
| --- | --- | --- |
| Cholestrol | **0.70**, 0.91 | 12.6, 19.4 |
| Lipids (-CH_3_) (LDL/VLDL) | 0.84, **0.87** | 23.4, 14.7 |
| Isoleucine | 0.93, **1.02**, 1.47 | 13.9, 14.6, 27.0 |
| Leucine | **0.97** | 24.8 |
| Valine | 0.98, **1.05** | 19.5, 21.1 |
| Isobutyrate | **1.15** | *b* |
| Lipids (CH_2_)_n_ (mainly LDL/VLDL) | 1.22, **1.30** | 32.7, 19.7 |
| Fucose | **1.20** | 18.3 |
| Lactate | **1.33**, 4.11 | 20.9, 69.2 |
| Alanine | **1.46** | 16.8 |
| Adipic acid | **1.58** | 28.8 |
| Arginine | **1.68** | 26.6 |
| Lysine | **1.72** | 29.4 |
| Acetate | **1.91** | 26.5/184.4 |
| Lipids (CH_2_–C= C) | **1.93** | *b* |
| Acetyl signals from glycoproteins | **2.05** | 23.1 |
| Proline | 2.07, **3.36** | 31.8, 48.9 |
| Glutamine | 2.08-**2.15** | 30.1 |
| Lipids (CH_2_–CO) | **2.25**-3.00 | 34.7 |
| Citrate | **2.50**-2.55 | 48.7, 78.2 |
| Lipids (CH= CH–CH_2_–CH= CH–) | 2.69, **2.71** | 26.2 |
| Albumin lysyl | **3.01** | 40.3 |
| Creatine | **3.04** | 39.5 |
| Choline | **3.22** | 55.0 |
| Taurine | **3.25** | 50.4 |
| Trimethyl N-oxide | **3.27** | *b* |
| Glucose and α-protons of amino acids | **3.3**-3.95 | 70.6- 76.7 |
| Myo-inositol | 3.56, **4.06** | 73.97, 74.94 |
| Glycerol | 3.56- **3.75** | 63.5 |
| Phenylalanine | 3.96, **7.3**- 7.44 | 58.94, 132.6, 131.3 |
| Creatinine | **3.98** | 59.0 |
| Glycerol of lipids | **5.23** | *b* |
| 3-hydroxybutyrate | **4.16** | 68.8 |
| Threonine | **4.26**- 4.30 | 69.0 |
| β-glucose | **4.75** | 99.0 |
| α-glucose | **5.26** | 94.8 |
| Lipids (–CH=CH–) | **5.30**-5.38 | 128.6 |
| Tyrosine | **6.90**, 7.22 | 119.2, 133.5 |
| Histidine | 7.03, **7.74** | 118.2, 136.2 |
| 1-methyl histidine | **7.78**, 7.82 | 141.12 |
| Formate | **8.46** | 151.8 |

*^b^* The signals were not determined. The bold signals were used for the generation of relative profile of groups in figure 6.

**Supplementary Table S3: Cross validated-ANOVA table for assessing the reliability of the generated models.**

| **ANOVA** |  |  |  |  |  |  |
| --- | --- | --- | --- | --- | --- | --- |
| ***Source of Variation*** | ***SS*** | ***df*** | ***MS*** | ***F*** | ***P-value*** | ***F crit*** |
| **Rows** | 5.25E+19 | 112 | 4.69E+17 | 1.993105 | 2.43E-09 | 1.230088 |
| **Columns** | 4.63E+22 | 249 | 1.86E+20 | 790.8973 | 0 | 1.152659 |
| **Error** | 6.56E+21 | 27888 | 2.35E+17 |  |  |  |
|  |  |  |  |  |  |  |
| **Total** | 5.29E+22 | 28249 |  |  |  |  |

**SS= sum of squares, DF= degree of freedom, MS= mean squares, F= F-test calculate value, p= p-value of the test, SD= standard deviation**


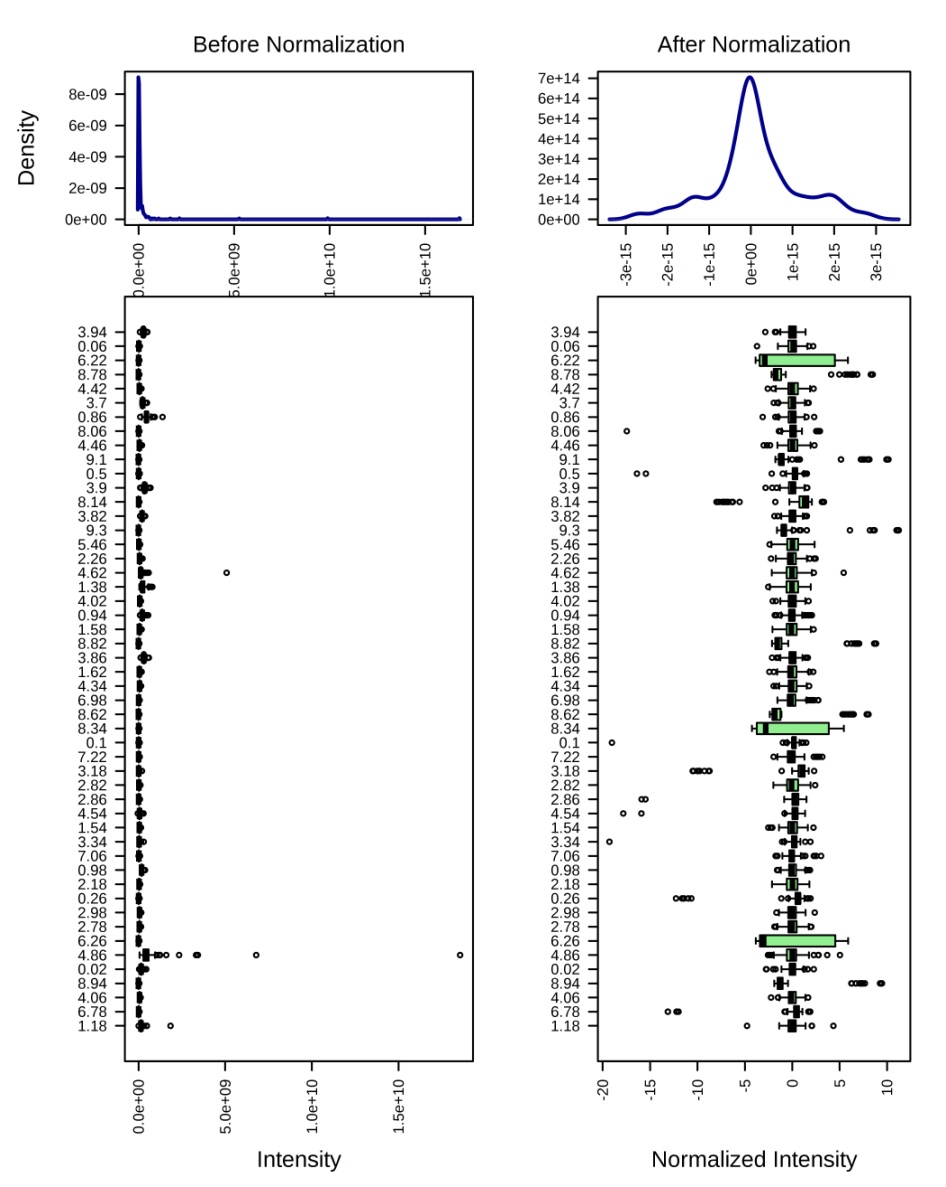

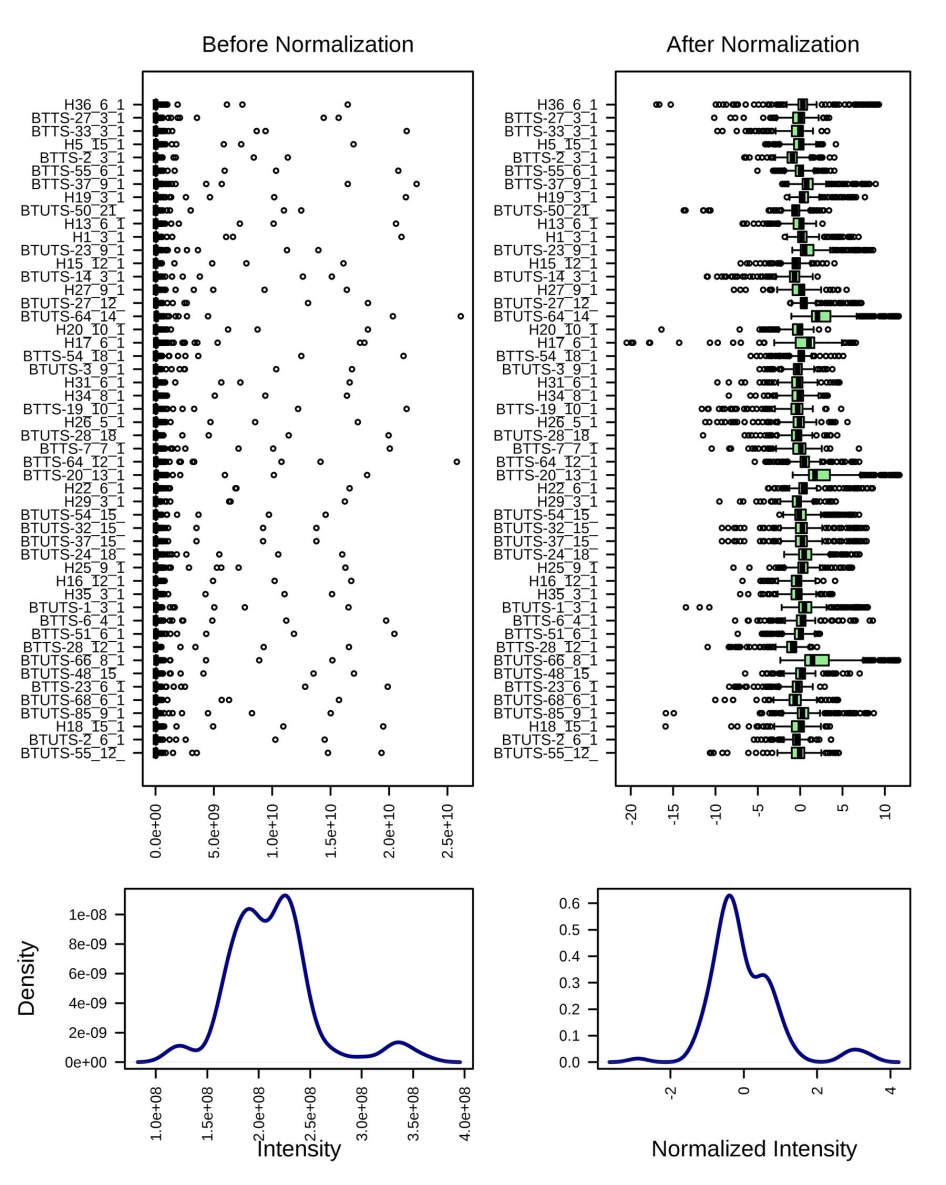


**A**

**B**

**Supplementary Figure S1:** Presentation of data (A) variables (B) samples, before and after normalization using unit area method.

**
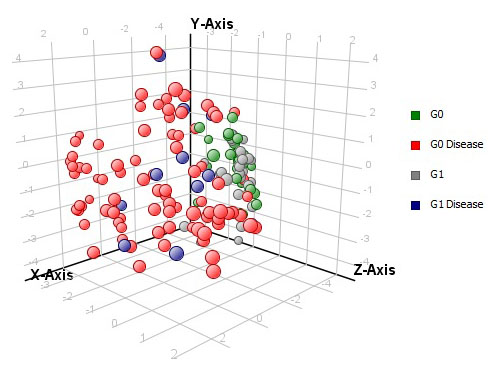
**

**Supplementary Figure S2:** PCA plot discrimination on the basis of age of samples [(GO: 0-20 years), (G1: 20-30 years)].

**
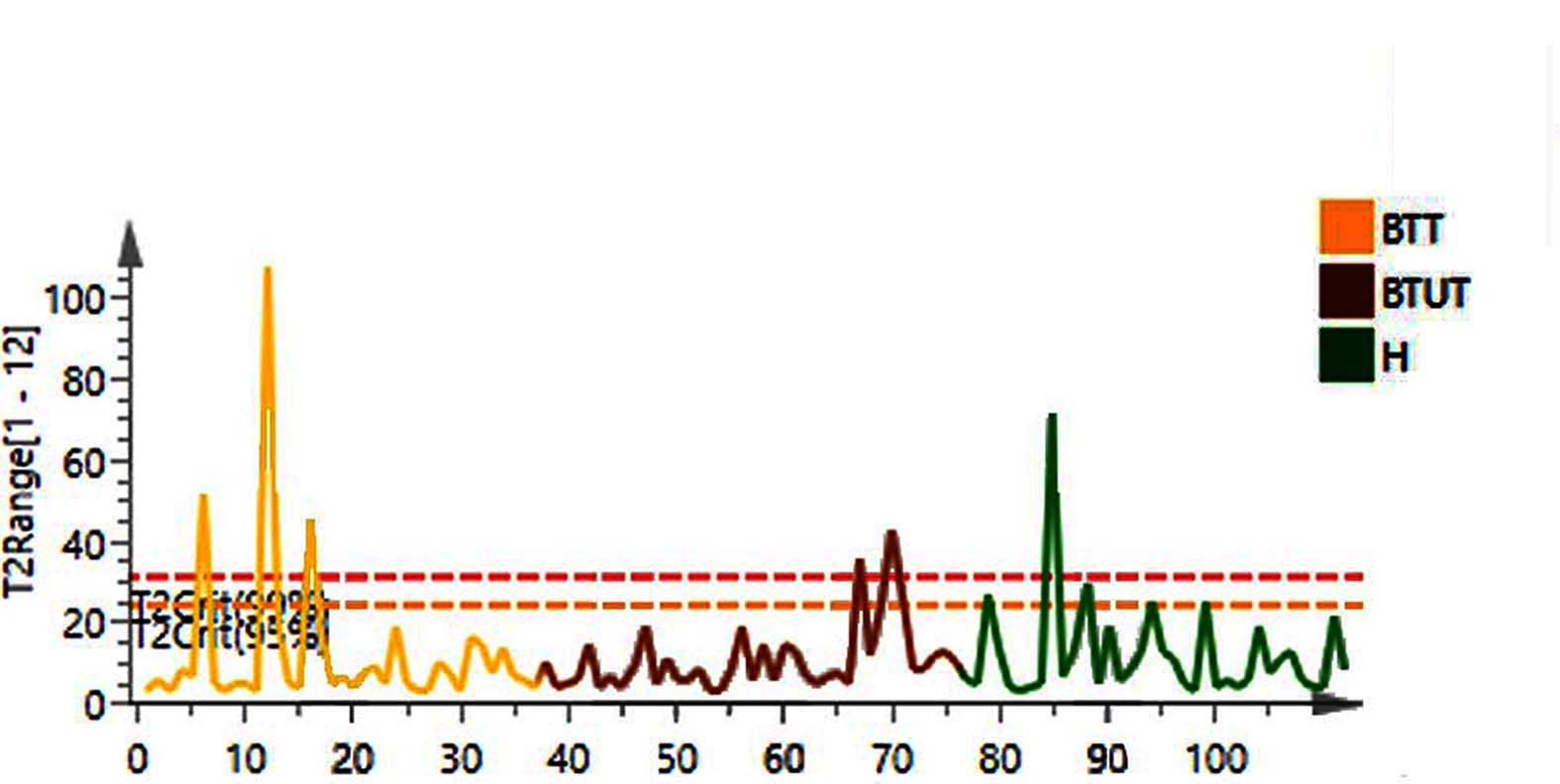
**

**Supplementary Figure S3:** Hotelling’s T2 plot with 99% and 95% confidence limits showing outliers. [Healthy (green), treated (yellow), untreated (red)].


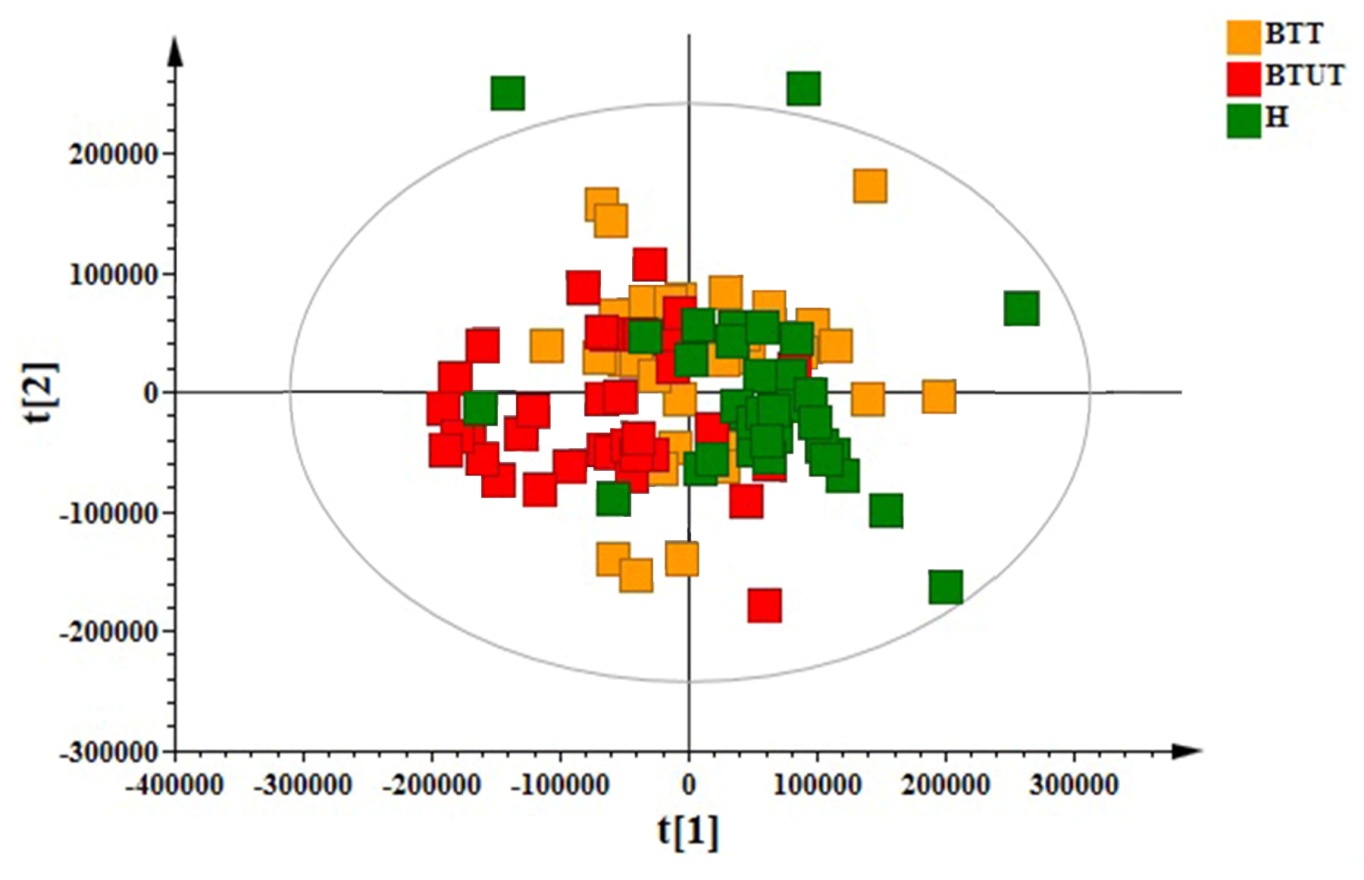


**Supplementary Figure S4:** PCA score scatter plot of ^1^H CPMG NMR spectra of serum from healthy (green), treated (yellow), untreated (red) β-thalassemia samples after excluding outliers.


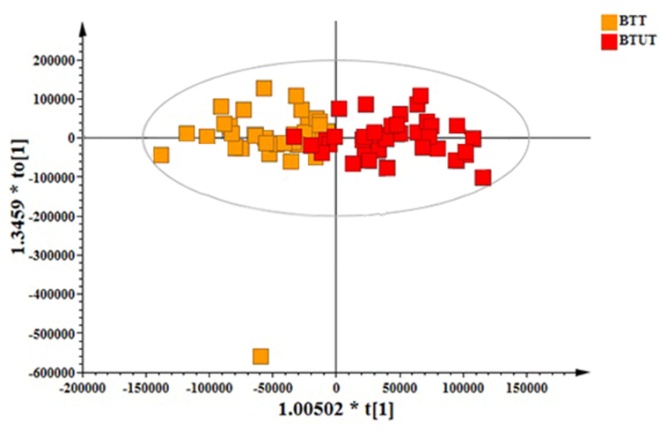

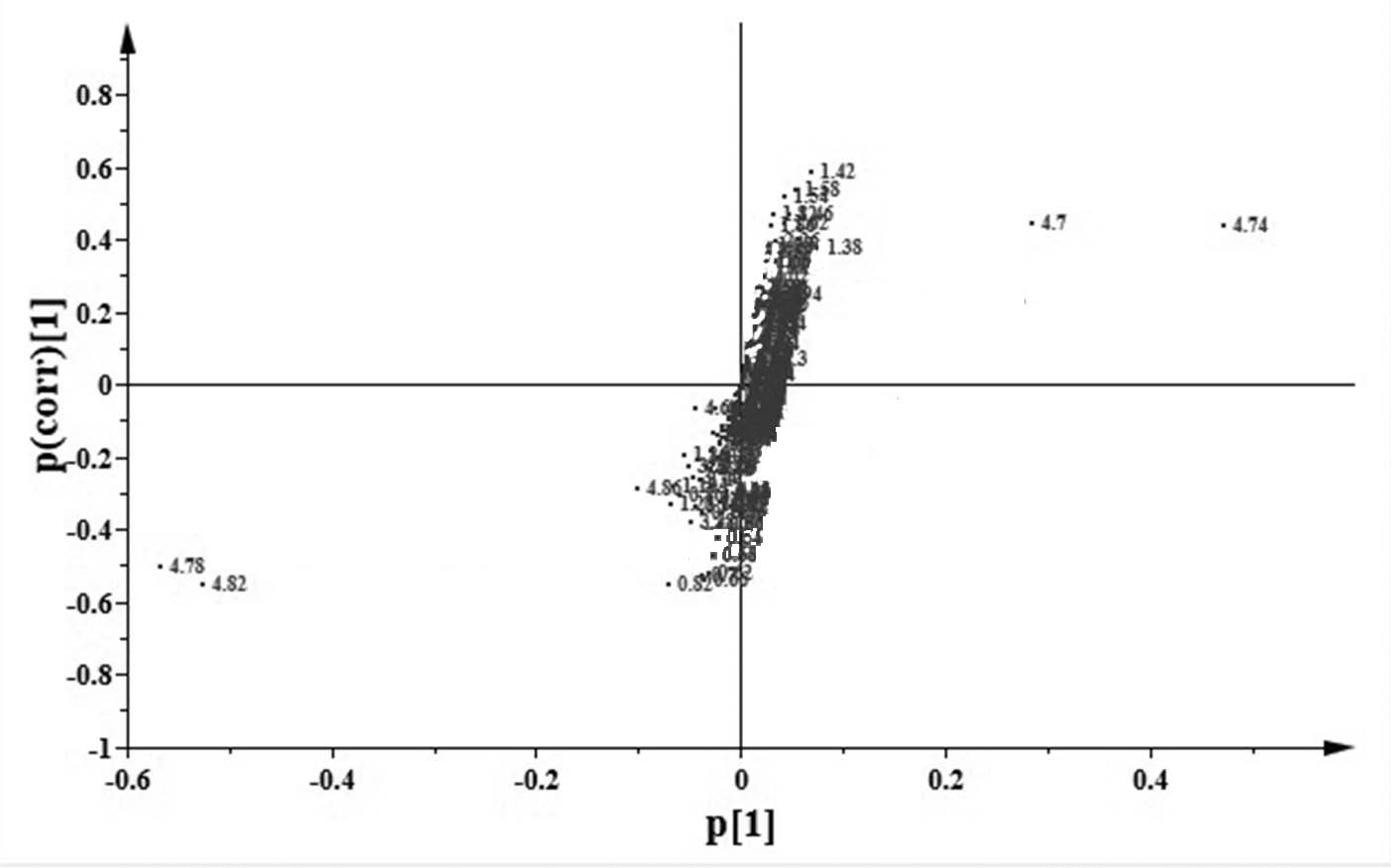


B

A


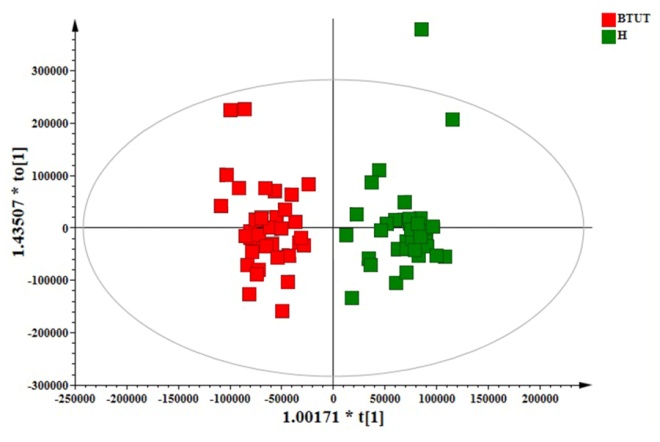

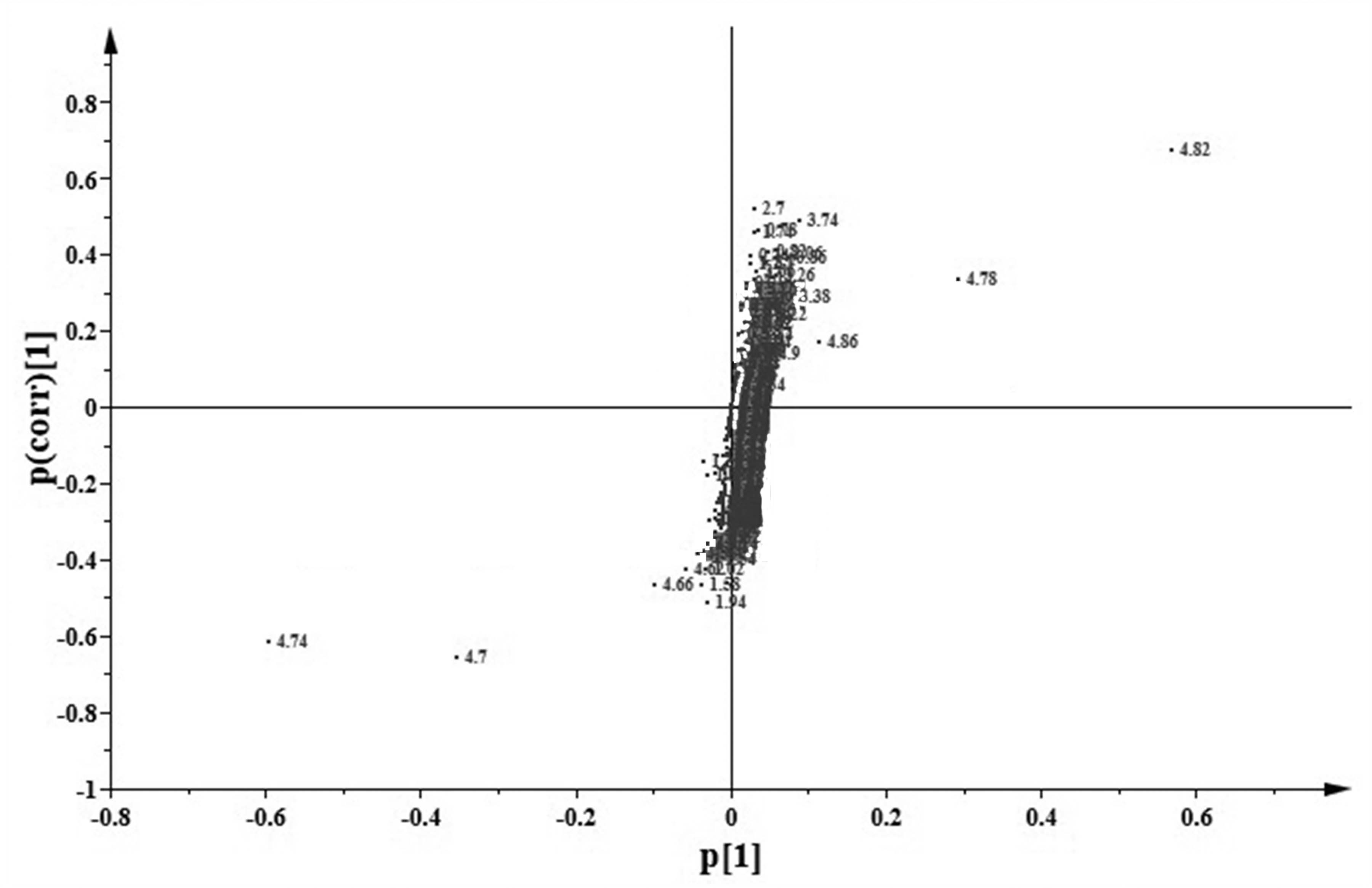


C

D


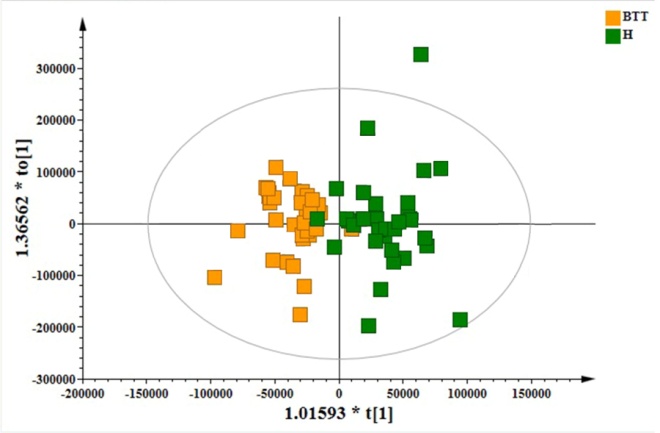

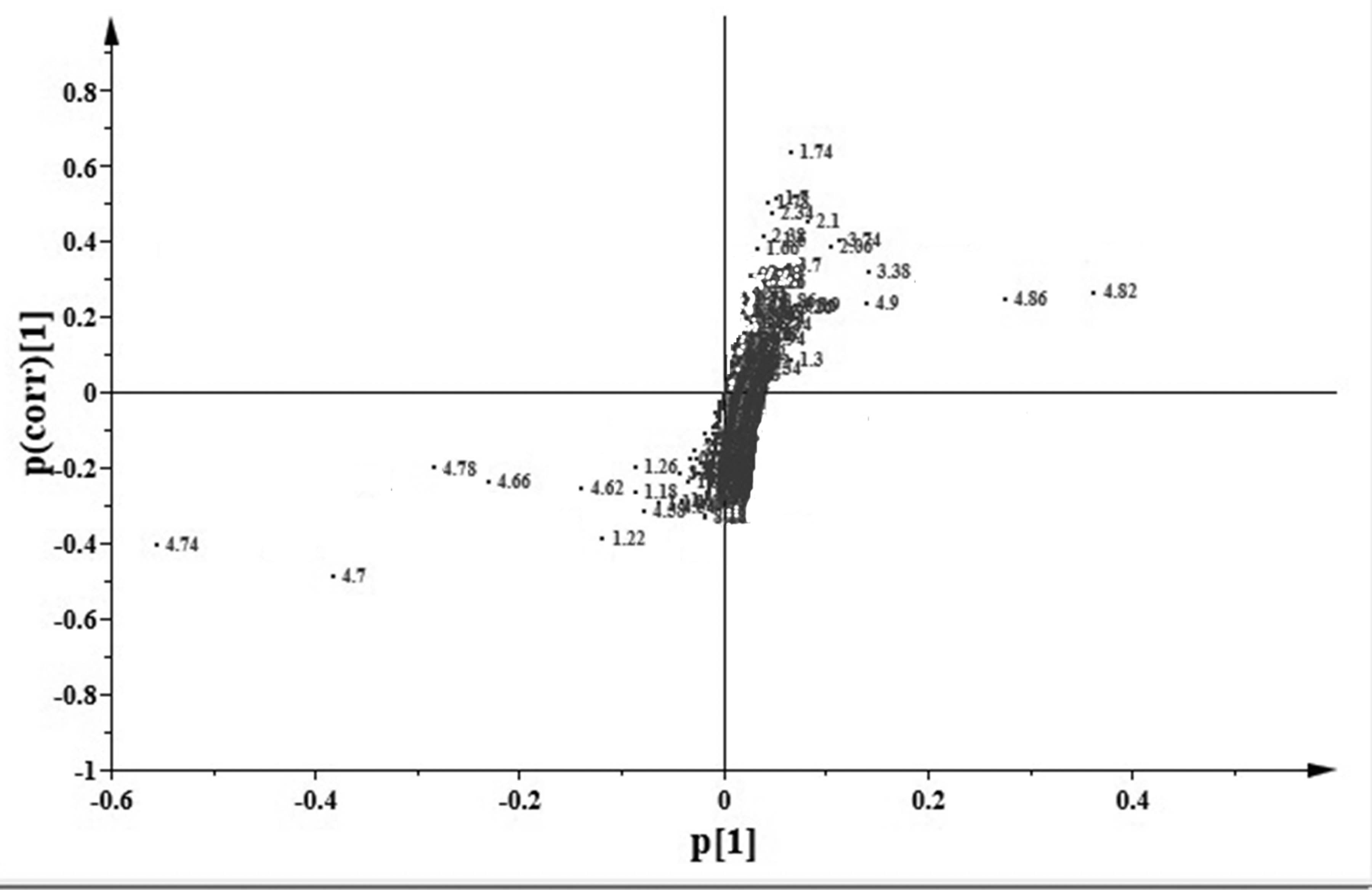


E

F

**Supplementary Figure S5:** Score scatter plot of OPLS-DA showing separation of pair of groups. [Healthy (green), treated (yellow), untreated (red)] with their corresponding S-plots.


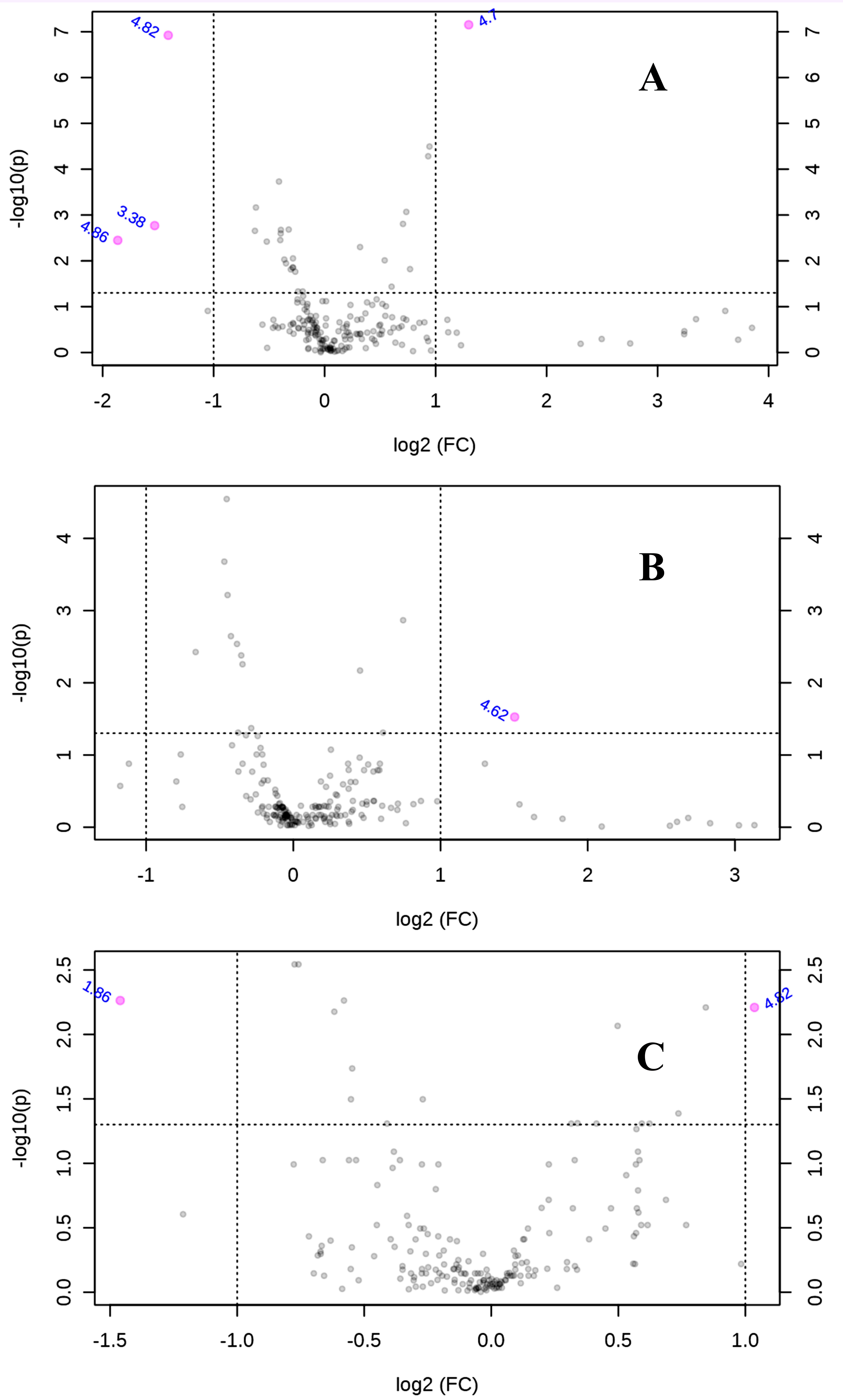


**Supplementary Figure S6:**  Volcano plots presenting fold change analysis on x-axis and p-values generated from Wilcoxon Rank test on y-axis. **(A)** BTUT *vs* H **(B)** BTT *vs* H **(C)** BTUT *vs* BTT

**
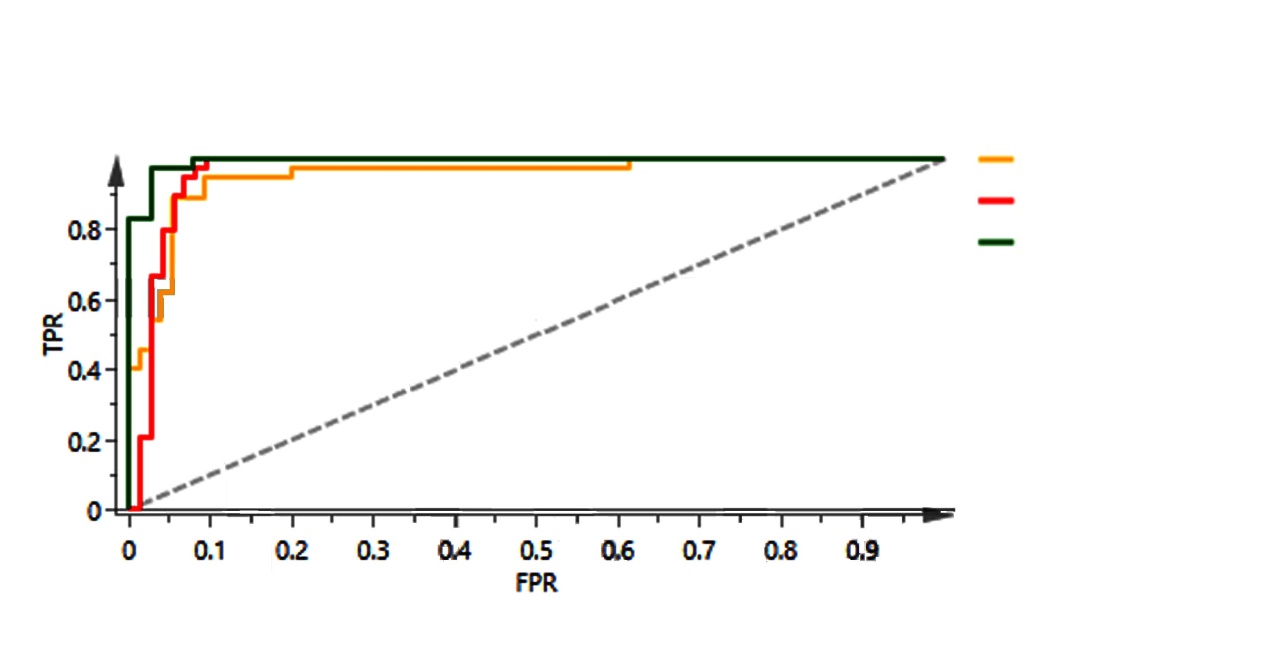
**

**Supplementary Figure S7:** Receiver operating characteristic (ROC) plot for OPLS-DA model showing sensitivity on y-axis and 1-specificity on x-axis [Healthy (green), treated (yellow), untreated (red)].

**
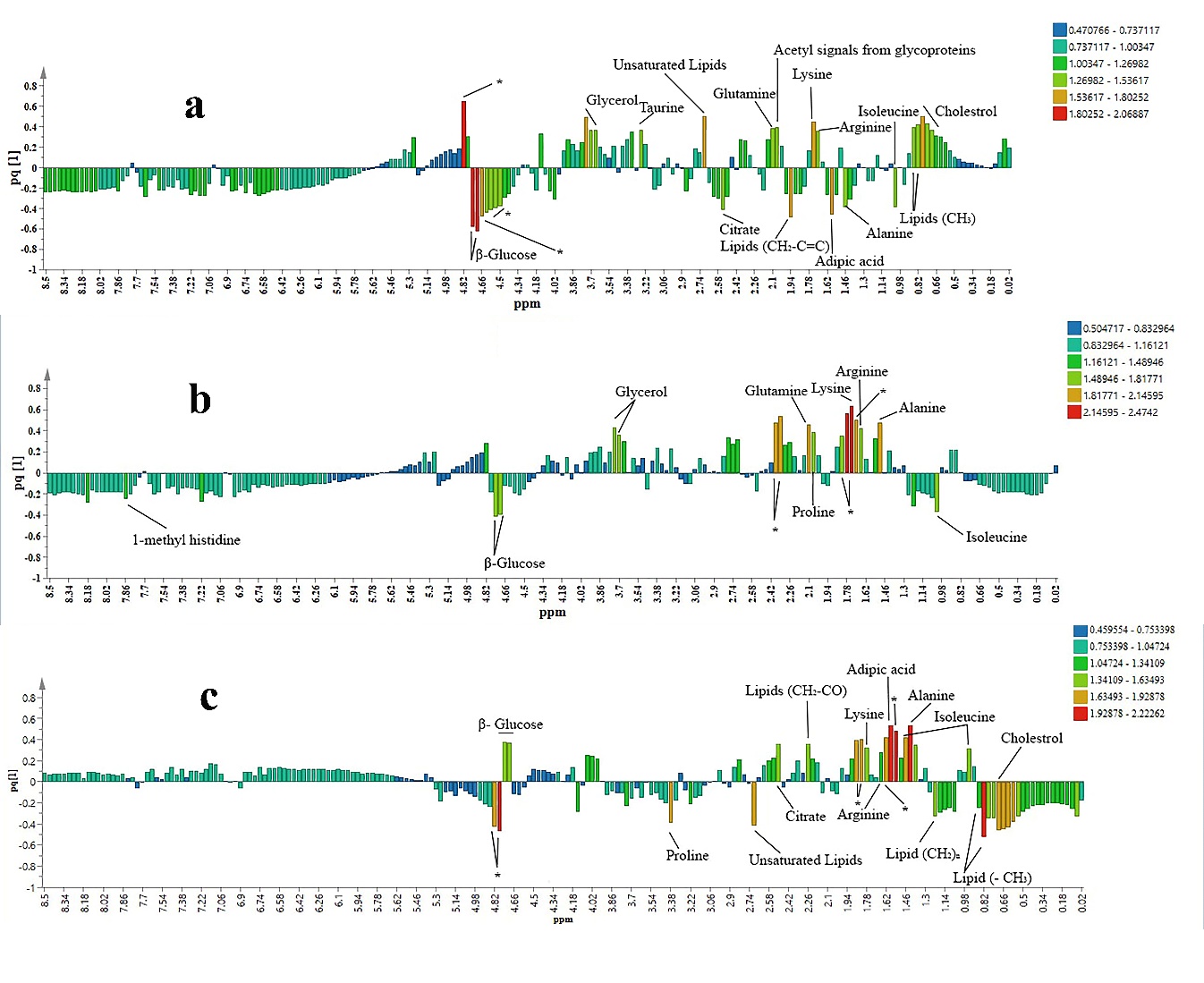
**

**Supplementary Figure S8: OPLS-DA loadings plot colored as a function of VIP between (a) BTUT and Healthy (b) BTT and Healthy and (c) BTUT and BTT.** Assignment of main signals having values (1-2) is indicated (unassigned signals with high VIP are marked with an asterisk).


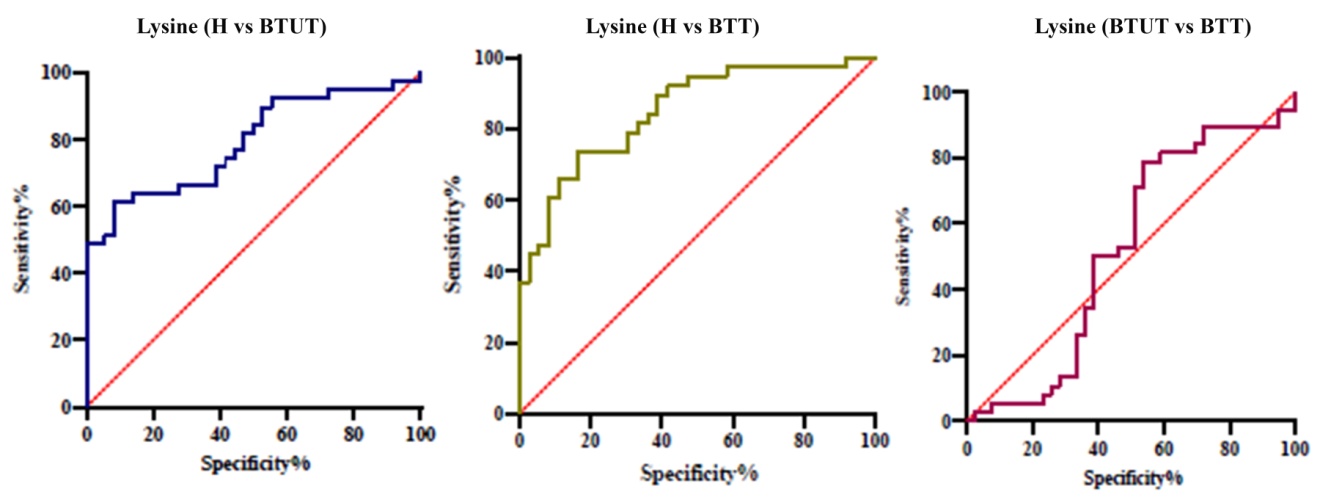

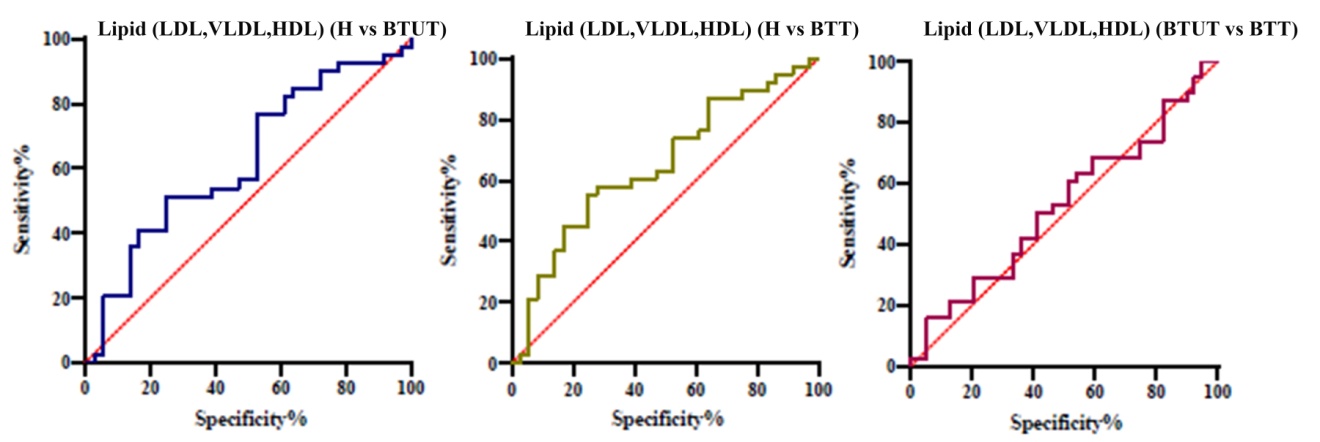

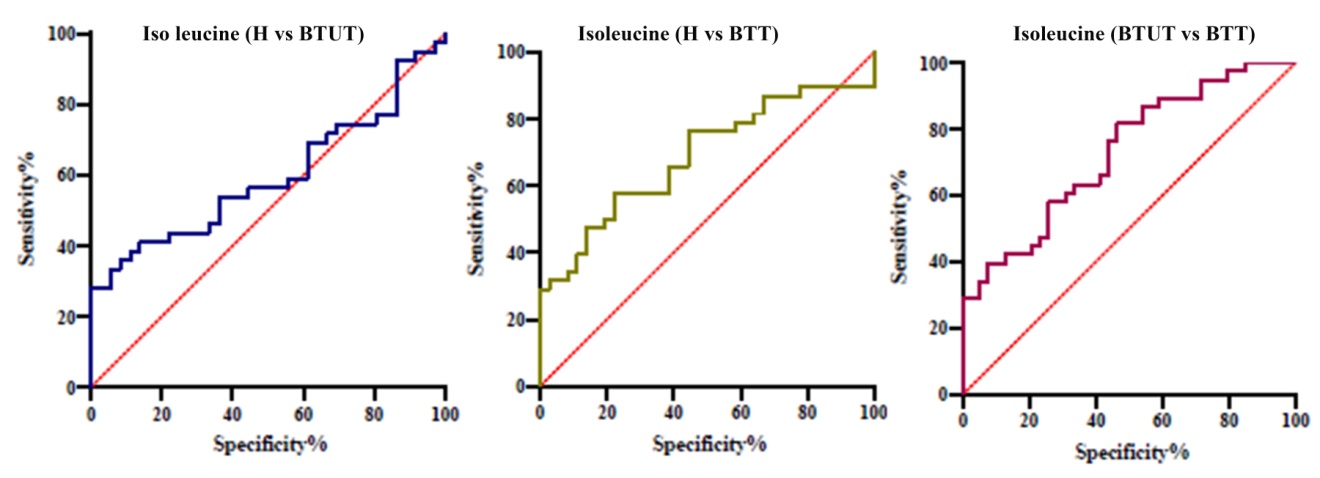

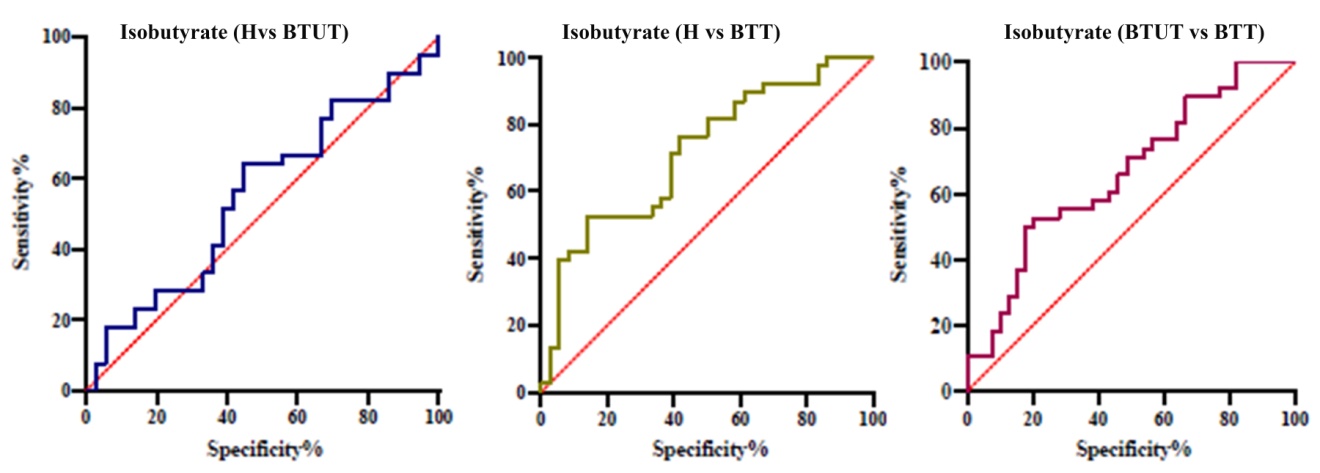

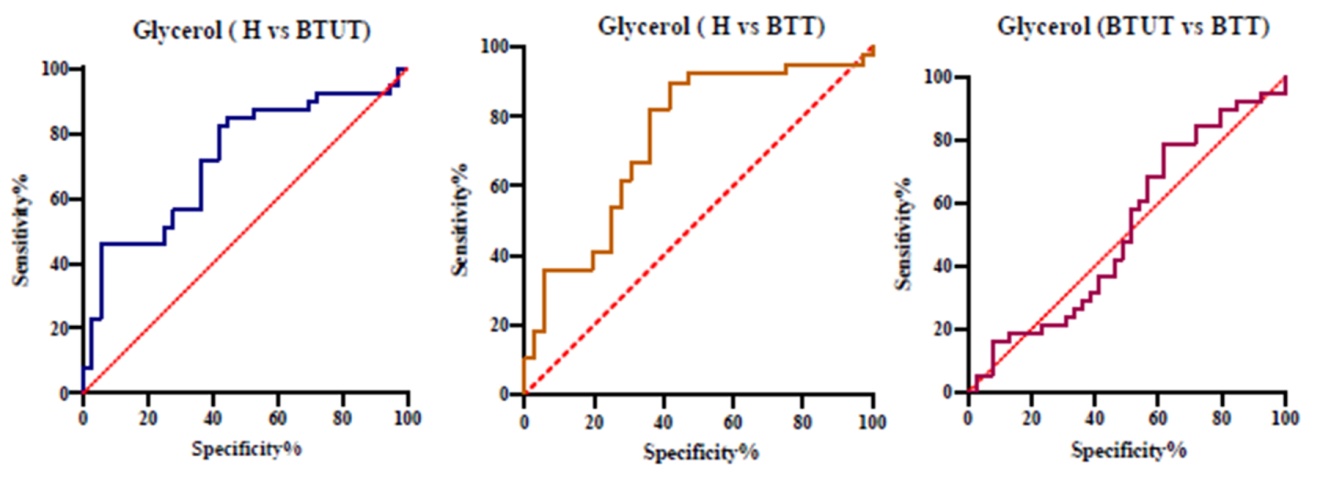

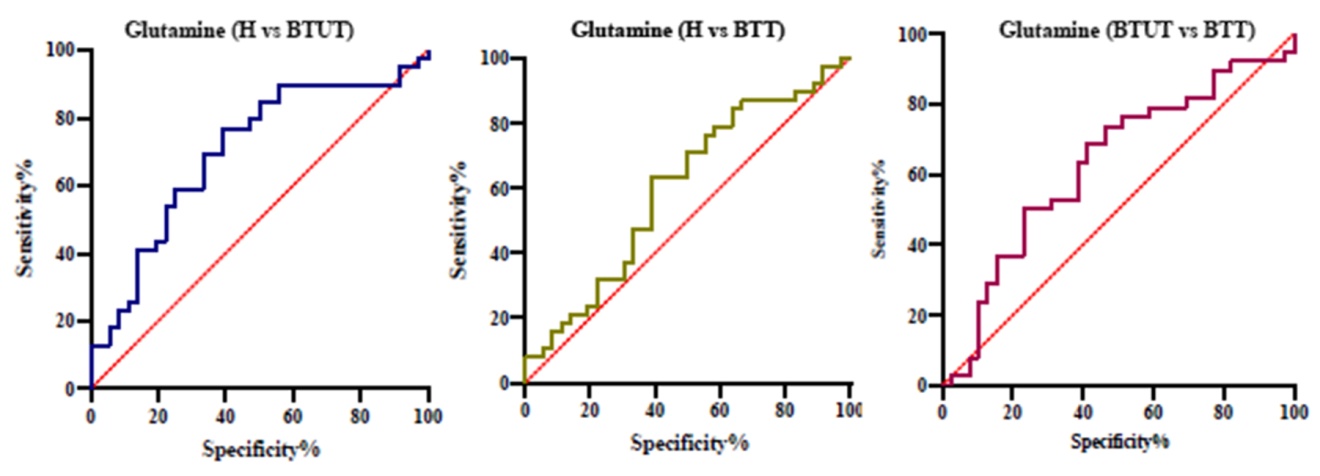

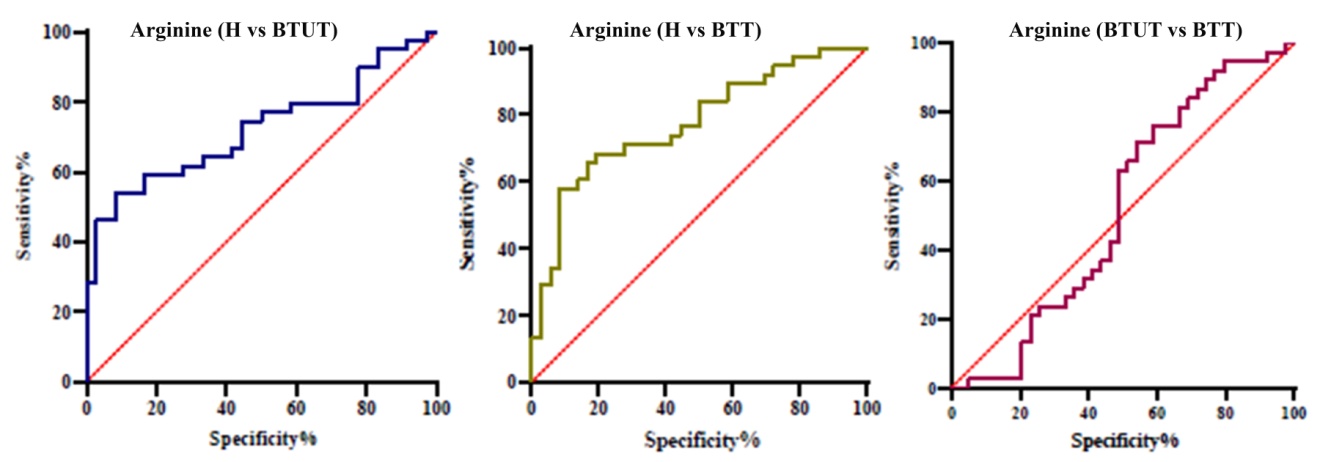

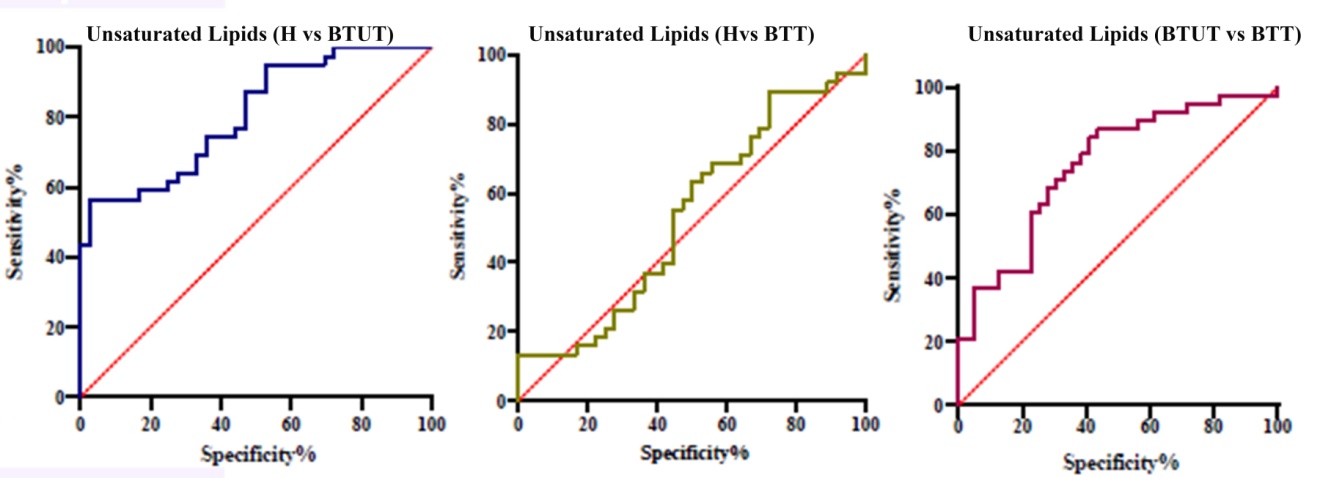


**Supplementary Figure S9:** Receiver operating characteristic (ROC) plot of selected discriminating metabolites.
